# Left Ventricular Assist Device: What the Internist Needs to Know. A Review of the Literature

**DOI:** 10.7759/cureus.4399

**Published:** 2019-04-05

**Authors:** Pranjal Boruah, Najam Saqib, Jumee Barooah, Dhiraj Baruah, Parikshit Sharma

**Affiliations:** 1 Cardiology, Geisinger Commonwealth School of Medicine, Wright Center for Graduate Medical Education, Scranton, USA; 2 Internal Medicine, Wright Center for Graduate Medical Education, Scranton, USA; 3 Radiology, Medical College of Wisconsin, Milwaukee, USA; 4 Cardiology, Rush University Medical Center, Chicago, USA

**Keywords:** heart failure (hf), left ventricular assist device (lvad), mechanical circulatory support (mcs), heart failure with reduced ejection fraction (hfref)

## Abstract

Left ventricular assist devices (LVADs) have revolutionized therapy for patients with Stage D heart failure (HF) with reduced systolic function providing not only improved survival benefits but also meaningful changes in quality of life and functional capacity. With technological advances and improved durability of devices, length of survival has significantly improved. With continued organ donor shortage, LVADs are frequently serving as a substitute for cardiac transplant as destination therapy, particularly among the elderly. Internists not only face the important challenge of identifying the patients in need referral for these advanced therapies, they are also faced with the challenges of taking care of these patients. This review will help the internists to better understand the present status, indications and advances in LVADs and also understand the complications and adverse effects associated with these devices.

## Introduction and background

The incidence and prevalence of patients with heart failure (HF) is increasing at alarming rates. HF has become one of the largest cardiovascular epidemics of modern times. With an aging population, advancement of therapies and improved survival of patients with HF, this is going to continue to be a major epidemic [1-3]. HF is a global problem with an estimated prevalence of 38 million patients worldwide and that number continues to rise. It is the most common diagnosis among hospitalized patients aged 65 years and in high-income nations. Despite the significant advancements in HF therapy, the prognosis of HF remains worse than that of most malignancies. Approximately 50% of the HF population has HF with reduced ejection fraction (HFrEF). A small subset of these patients (0.5-5%) with HF respond poorly to standard guideline directed medical therapy (GDMT) and progress to chronic advanced HF [4,5]. Among patients with HF, those with advanced age and higher co-morbidities (due to the extremely poor prognosis), palliative care had been the only option. Even though heart transplantation is an excellent treatment option for patients who are good candidates, the availability of suitable donor remains a limiting factor.

Patients with Stage D HF (advanced HF) have a poor short-term survival and have a very small chance of receiving a transplant. It is for this patient population that the emergence of continuous flow (CF) left ventricular assist devices (LVADs) holds the greatest promise. It does so by augmenting the circulation to meet the body’s physiological needs thereby improving survival and quality of life.

The American Heart Association (AHA)/American College of Cardiology (ACC) guidelines for 2013 provide a Class IIa Level of recommendation (Level of Evidence: B) for LVAD therapy among a selected subgroup of patients [left ventricular ejection fraction (LVEF) < 25%, New York Heart Association (NYHA) Class III-IV functional status despite GDMT, including cardiac resynchronization therapy (CRT) when indicated with either high predicted 1-2 year mortality or dependence of continuous parenteral inotropic support] in whom definite management like cardiac transplantation or cardiac recovery is anticipated. It also suggests that the use of nondurable mechanical circulatory support (MCS), including the use of percutaneous and extracorporeal ventricular assist devices (VADs), is reasonable as a “bridge to recovery” or “bridge to decision” for carefully selected patients with HFrEF with acute, profound hemodynamic compromise (Level of Evidence: B) while durable MCS is reasonable to prolong survival for carefully selected patients with Stage D HFrEF (Level of Evidence: B) [6]. The focused update of the ACC/AHA guidelines from 2017 reflects no changes to these recommendations [7].

## Review

Stage D heart failure and treatment: emerging role of LVADs

Various terminologies have been used to describe the group of patients who are classified with ACC/AHA stage D HF, including “advanced HF,” “end-stage HF,” and “refractory HF.” In the 2009 ACCF/AHA HF guideline, stage D was defined as “patients with truly refractory HF who might be eligible for specialized, advanced treatment strategies, such as MCS, procedures to facilitate fluid removal, continuous inotropic infusions, or cardiac transplantation or other innovative or experimental surgical procedures, or for end-of-life care, such as hospice.” The European Society of Cardiology has developed a definition of advanced HF with objective criteria (Table 1) [8]. There are clinical clues that may assist clinicians in identifying patients who are progressing toward advanced HF (Table 2) [9]. The Interagency Registry for Mechanically Assisted Circulatory Support (INTERMACS) has developed seven profiles that further stratify patients with advanced HF (Table 3) [10].

**Table 1:** ESC definition of advanced HF. BNP: B-type natriuretic peptide; CRT: Cardiac resynchronization therapy; ESC: European Society of Cardiology; GDMT: Guideline-directed medical therapy; HF: Heart failure; LVEF: Left ventricular ejection fraction; NT-proBNP: N-terminal pro-B-type natriuretic peptide; NYHA: New York Heart Association; PA: Pulmonary artery; PCWP: Pulmonary capillary wedge pressure; RAP: Right atrial pressure. Adapted from Metra et al.

1) Severe symptoms of HF with dyspnea and/or fatigue at rest or with minimal exertion (NYHA class III or IV)
2) Episodes of fluid retention (pulmonary and/or systemic congestion, peripheral edema) and/or reduced cardiac output at rest (peripheral hypoperfusion)
3) Objective evidence of severe cardiac dysfunction shown by at least one of the following: a) LVEF < 30%. b) Pseudonormal or restrictive mitral inflow pattern. c) Mean PCWP > 16 mm Hg and/or RAP > 12 mm Hg by PA catheterization. d) High BNP or NT-proBNP plasma levels in the absence of noncardiac cause
4) Severe impairment of functional capacity shown by one of the following: a) Inability to exercise. b) Six-minute walk distance 300 m. c) Peak V· O2 < 12 to 14 mL/kg/min
5) History of HF hospitalization in past six months
6) Presence of all the previous features despite “attempts to optimize” therapy, including diuretics and GDMT, unless these are poorly tolerated or contraindicated, and CRT when indicated.

**Table 2:** Clinical events and findings useful for identifying patients with advanced HF. ACE: Angiotensin-converting enzyme; BUN: Blood urea nitrogen; ED: Emergency department; HF: Heart failure; ICD: Implantable cardioverter defibrillator. Adapted from Russell et al.

Repeated hospitalizations or ED visits for HF in the past year
Progressive deterioration in renal function (e.g., rise in BUN and creatinine)
Weight loss without other cause (e.g., cardiac cachexia)
Intolerance to ACE inhibitors due to hypotension and/or worsening renal function
Intolerance to beta blockers due to worsening HF or hypotension
Frequent systolic blood pressure <90 mm Hg
Persistent dyspnea with dressing or bathing requiring rest
Inability to walk 1 block on the level ground due to dyspnea or fatigue
Recent need to escalate diuretics to maintain volume status, often reaching daily furosemide equivalent dose >160 mg/d and/or use of supplemental metolazone therapy
Progressive decline in serum sodium, usually to <133 mEq/L
Frequent ICD shocks

**Table 3:** INTERMACS: Profiles for patient selection. NYHA: New York Heart Association Classification, INTERMACS: Interagency Registry for Mechanically Assisted Circulatory Support. Adapted from Stevenson et al.

		Possible profile modifier		
Profile	Description	Temporary circulatory support (TCS)	Arrhythmia (A)	Frequent flyers (FF)
1	Critical cardiogenic shock	X	X	
2	Progressive decline on inotropic support	X	X	
3	Stable but inotrope dependent	X (In hosp)	X	X
4	Resting symptoms home on oral therapy		X	X (if home)
5	Exertion intolerant		X	X
6	Exertion limited		X	X
7	Advanced NYHA Class III symptoms		X	

Cardiac transplantation has been associated with excellent outcomes with a median survival of 10.7 years and survival, conditional on surviving to one year after transplant, reaching 13.6 years [11]. It also leads to significant improvement in the quality of life (QOL). In the United States, the number of candidates actively awaiting heart transplant increased dramatically from 2005 to 2019 by more than 300%. In January 2019, the number of patients waiting for a cardiac transplant was 3821 (as opposed to 1262 in 2005), suggestive of the fact that transplant rates have not increased at the same rate as listings [12]. The major limiting factor to the growth of the cardiac transplant program has been the limited donor supply. With the results of the PROCEED II (Randomized Study of Organ Care System Cardiac for Preservation of Donated Hearts for Eventual Transplantation) trial which has shown non-inferiority of ex-vivo preservation to cold ischemia undergoing cardiac transplant with standard donors, the geographic limit with cold preservation techniques may be a thing of the past [13]. Nevertheless, despite the promise of more usable organs, with the donor supply remaining flat, cardiac transplantation is unfortunately not the solution for the majority of the patients.

**Figure 1:**
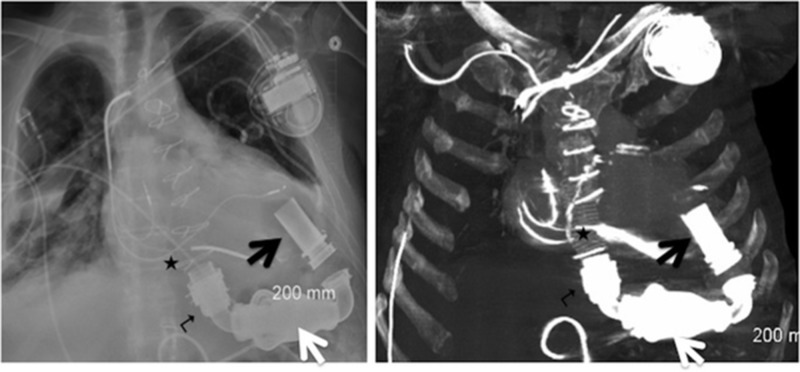
Plain radiograph and maximum intensity projection (MIP) computed tomography (CT) images showing Heartmate II left ventricular assist device (LVAD). Black arrow – inflow cannula; white arrow – pump; turn arrow – band relief; black star – outflow cannula.

**Figure 2:**
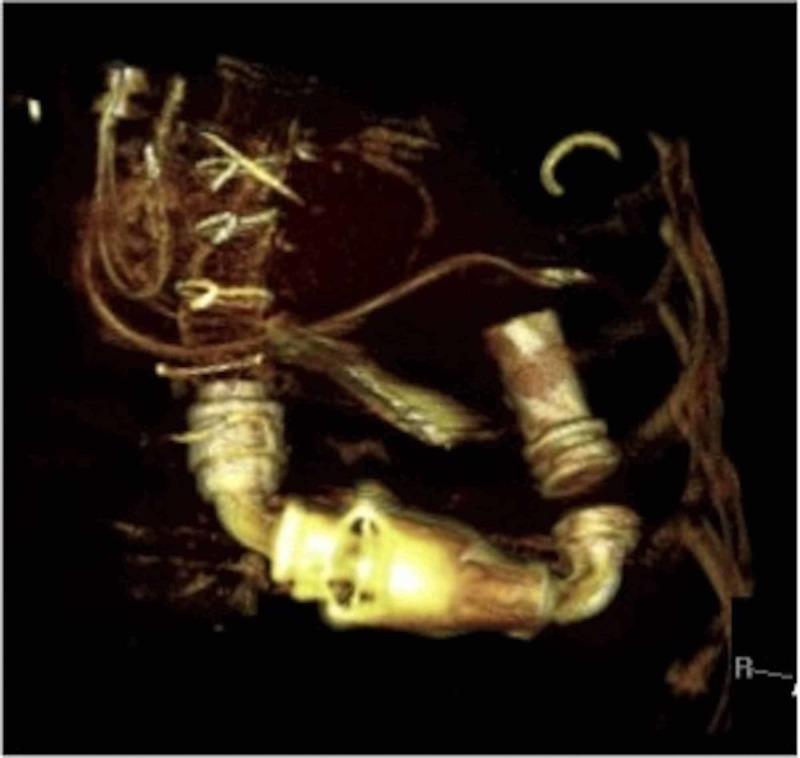
Volume rendered 3D computed tomography (CT) image showing parts of Heartmate II left ventricular assist device (LVAD) as described in Figure 1.

Fortunately, with the advent of MCS, transplant patients are able to receive mechanical support while they wait for an acceptable organ. LVADs are typically offered to transplant candidates who are developing end-organ damage despite maximal medical therapy with an anticipated long waitlist time (large size and/or blood type O recipient) (Figure 1) [14]. These categories correspond to INTERMACS Level 1-3. The INTERMACS scale assigns patients with advanced HF into seven levels according to hemodynamic profile and functional capacity. Initial use of pulsatile-flow LVADs and total artificial hearts demonstrated an improved survival among patients with advanced HF treated with Bridge to Transplant (BTT). The REMATCH study was the landmark study published in 2001 that demonstrated improved survival in advanced HF patients ineligible for transplantation treated with LVAD vs. optimal medical therapy [15]. More recent studies have shown continued improvement in survival and quality of life in patients implanted with CF LVADs compared with first-generation pulsatile devices (Table 4). The HM II was the first CF LVAD to receive US Food and Drug Administration approval for commercial use as BTT therapy for patients with advanced HF waiting for a heart transplant. CF devices are now predominantly used for BTT [16]. Recent registry data demonstrate that the use of the HM II as BTT has increased since 2008, and in 2011 about 30% of patients at the time of listing were implanted with an HM II. The annual mortality of status 1A and 1B patients on the United Network of Organ Sharing (UNOS) waiting list has decreased in recent years, which correlates with an increase in HM II use. With the recently published data from MOMENTUM 3, which was primarily designed to test the non-inferiority of the HeartMate 3 LV assist system to the HeartMate II LVAD (Figure 2), there were better outcomes at six months than implantation of an axial-flow pump, primarily because of the lower rate of reoperation for pump malfunction [17].

**Table 4:** Studies reporting continued improvement in survival and quality of life in patients implanted with CF LVADs compared with first-generation pulsatile devices. DT: Destination therapy; ENDURANCE: A prospective, randomized, controlled, un-blinded, multi-center clinical trial to evaluate the HeartWare" ventricular assist system (VAS) for destination therapy of advanced heart failure; HM3: HeartMate3; HMII: HeartMate II; HMVE: HeartMate VE; INTERMACS: Interagency registry for mechanically assisted circulation; INTrEPID: Investigation of nontransplant-eligible patients who are inotrope dependent; LVAD: Left ventricular assist device; MOMENTUM 3: Multicenter study of MagLev technology in patients undergoing mechanical circulatory support therapy with HeartMate3; REMATCH: Randomized evaluation of mechanical assistance for the treatment of congestive heart failure; ROADMAP: Risk assessment and comparative effectiveness of left ventricular assist device and medical management in ambulatory heart failure patients; HMXVE: HeartMate XVE; LVEF: Left ventricular ejection fraction; COPD: Chronic obstructive airway disease; BMI: Body mass index.

Study	Number of Patients	Device Tested	Comparison Group	Design	Patient Characteristics	Exclusion Criteria	Primary Outcome Freedom from Primary Outcome
REMATCH	129	HMXVE	Medical Therapy	Prospective 1:1 HeartMate XVE vs. medical therapy	NYHA Functional Class IV for 60 days, LVEF < 25% and peak oxygen consumption < 14 ml/min/kg (unless on balloon pump or physically unable to perform exercise test) or intra-aortic balloon pump or IV inotrope dependent for 14 days	Patient cannot be enrolled in a clinical trial with mortality as an end point. No investigational agent within 30 days of randomization. Must not have undergone cardiomyoplasty or ventricular reduction operation.	Death from any cause. One year HMXVE 52%, medical therapy 25%. P = 0.001
INTrEPID	55	NovaCor	Medical Therapy	Prospective Nonrandomized	LVEF < 25% for ≥ 6 months, NYHA Class IV symptoms for ≥ 3 months, two failed attempts at weaning from inotropic support by at least seven days	Body surface area <1.5 m^2^. Contraindication for anticoagulation, Presence of mechanical aortic valve, CVA or TIA within six months of enrollment, >70% carotid stenosis or ulcerated carotid plaque, drug or alcohol dependency, systemic infection, serum creatinine > 5.0 mg/dl, mechanical ventilator support for >48 hrs at time of enrollment. Life expectancy < 2 yrs	All cause mortality at six months, 1 yr: Novocor 27%, Medical Therapy 11%. P = 0.02
HeartMate II DT Trial	192	HeartMate II	HMXVE	Prospective randomized 2:1 HeartMate II vs. HMXVE	LVEF < 25%, NYHA Class IIB or IV symptoms for at least 45 days of 60 days before enrollment, dependent on IABP for a period of seven days or inotropes for a period of at least 14 days before enrollment, peak oxygen consumption <14 ml/min/kg or less than 50% of predicted value	Irreversible severe renal, pulmonary or hepatic dysfunction or active infection	Survival at two years of disabling stroke and device replacement HM II 46%, HMXVE 11%. P < 0.001
Early vs. Late HM II DT	Mid trial n = 281 vs. Early trial n = 133	HeartMate II	HMXVE	Retrospective analysis of patients enrolled in HeartMate II DT Trial			Survival at two years of disabling stroke and device replacement Mid trial 59%; Early trial 50%; P = 0.073
HM II Post-approval	247	HeartMate II	HMXVE	Prospective evaluation of the first 247 patients who underwent HMII implantation after FDA approval vs. historical control group		Patients preoperatively identified for destination therapy in INTERMACS	Survival at two years of disabling stroke and device replacement. Post approval 54%, HMII DT Trial 44%; P = 0.042
ENDURANCE	446	HeartWare HVAD	HMII	Prospective 2:1 randomization	LVEF < 25%, NYHA Class IIB or IV symptoms for at least 45 days of 60 days before enrollment, dependent on IABP for a period of seven days or inotropes for a period of at least 14 days before enrollment, body surface area ≥1.2 m^2^.	BMI > 40, prior cardiac transplant, eligible for cardiac transplant, history of untreated abdominal/thoracic aneurysm >5 cm, cardiothoracic surgery within 30 days, acute myocardial infarction within 14 days, symptomatic cerebrovascular disease, or stroke, severe RV failure, active uncontrolled infection, serum creatinine >3.0 or requiring dialysis, contraindications for anticoagulation, cirrhosis of liver or AST/ALT > 3 times upper limit	Survival at two years of disabling stroke and device replacement HVAD 55%; HM II 57.4%; P = 0.67
ROADMAP	200	HMII	Medical Therapy	Observational study of DT in INTERMACS profile 4-7	Age 18-85 yrs, NHYA Class IIIB or IV functional limitations, and LVEF ≤ 25% on optimal medical therapy. Inability to tolerate neurohormonal antagonist, 6 min walk distance <300 m within 45 days before enrollment, One unscheduled hospitalization for HF in last 12 months	Presence of mechanical aortic or mitral valve including planned conversion to bioprosthesis, platelet count < 100,000/ml, inability to perform 6 min walk test, IV inotrope within 45 days, existence of any MCS pregnancy, history of cardiac/other organ transplant, psychiatric disease, active uncontrolled infection, intolerance to anticoagulation, coronary revascularization within three months, GFR, 25 ml/min or need for renal replacement therapy, any condition that could limit survival to less than two years	Survival on original device and increase in 6 min walk ≥ 75 m at one year. HMII 39%; medical therapy 21%; P = 0.012
INTERMACS	3243	Continuous flow devices implanted as DT		DT patients entered into INTERMACS			Survival one year 75%; three years 57%
MOMENTUM 3	294	HM3	HMII	1:1 Randomization	Age ≥ 18 yrs, BSA ≥ 1.2 m^2^, LVEF ≤ 25%, Inotrope dependent or Cardiac Index (CI) < 2,2 L/min/m^2^ for at least 45 days of 60 days before enrollment on optimal medical management	High surgical risk, HF secondary to uncorrected thyroid disease, obstructive cardiomyopathy, pericardial disease, amyloidosis or restrictive cardiomyopathy, pregnancy, history of organ transplant, psychiatric disease, active uncontrolled infection, intolerance to anticoagulation, severe COPD, history of stroke, serum creatinine >2.5 mf/dl or need for dialysis, untreated AAA. 5.0 cm within six months, any condition that could limit survival to less than two years	Survival-free of disabling stroke on original device six months, HM 3 86.2%, HMII 76.8%. P < 0.001

Indications for mechanical circulatory support

There are four major indications for the use of LVADs:

1. Bridge to destination: this indication is for heart transplant patients who are either too sick to wait for the donor to be identified because of severe acute or acute on chronic HF, or have contraindications (that are deemed transient) to transplantation.

2. Destination therapy: LVADs are used as lifelong support as an alternative for transplantation for patients deemed ineligible for heart transplantation.

3. Bridge to myocardial recovery.

4. Bridge to bridge: this is for patients who present with severe shock or following a cardiac arrest and are supported with a temporary support VAD to see if they become candidates for long-term support devices.

The most common indication, which constitutes 40% of all LVAD implantation according to data from INTERMACS, is for a bridge to destination therapy [17]. It is used when the best long-term option for a given patient is unclear at the time of LVAD implantation.

Data from the ISHLT (International Society of Heart and Lung Transplantation) registry show more than 33% of all patients who underwent transplantation have an LVAD [18,19]. This percentage can vary according to countries and can be as high as 75% in programs where donor availability is low. Duration of mechanical support does not seem to have an adverse impact on mortality after cardiac transplant.

Inclusion/exclusion criteria and patient selection for LVADs

Centers for Medicare and Medicaid Services have established criteria for implantation of LVAD which are derived from the REMATCH (Randomized Evaluation of Mechanical Assistance for the Treatment of Congestive Heart Failure) and HeartMate II destination therapy (DT) trials [20].

1. Patients with NYHA functional Class IV symptoms who have failed to respond to optimal medical management, for at least 45 of the past 60 days, or have been intra-aortic balloon pump (IABP) dependent for seven days or IV inotrope dependent for 14 days.

2. Left ventricular ejection fraction of <25%, and

3. The functional limitation with a peak oxygen consumption of <14 ml/kg/min, unless on an IABP, IV inotrope, or physically unable to perform the exercise test.

Absolute contraindications include systemic illness with a life expectancy of fewer than two years or malignancy within five years, irreversible renal and hepatic dysfunction, severe obstructive pulmonary disease or other systemic diseases with multi-organ involvement [21]. However, LVADs may be an acceptable option for patients with recent cancer, which might theoretically be cured, but unlikely to have a five-year disease-free survival typically required for cardiac transplantation. Active infection with HIV or advanced end organ function such as serum creatinine of 3.0 mg/dL may not preclude patients from LVAD implantation [22].

Presence of bleeding diathesis may be a serious contraindication for LVAD unless coagulopathy is caused by reversible hepatic dysfunction. Bleeding is one of the most common complications of LVAD with a four-fold increased risk of reoperation over standard open-heart surgery [23]. The most common site of bleeding is in the upper gastrointestinal (GI) tract and is typically caused by or associated with development of arterial-venous malformations, primarily located in the stomach and early portions of the small bowel [24-26]. Recent attention has been directed at the uniform reduction in multimers of von Willebrand factor in the serum in response to non-pulsatile flow as an explanation for the increased bleeding association with continuous flow VADs [27,28].

Development of right ventricular (RV) failure is associated with the worse outcome if it develops after LVAD implantation. It is associated with higher mortality, greater risk of bleeding and/or re-operation, longer hospitalization and a higher rate of renal insufficiency [29-31]. The risk of RV failure occurs in up to 20% of patients especially in the setting of biventricular dysfunction in non-ischemic cardiomyopathy.

Moreover, all VAD patients should undergo a psychosocial evaluation by a trained mental health professional and social workers to ensure that are able to receive adequate postoperative care and medications before any decision for VAD implantation. Psychosocial predictors of poor post-implant outcomes are mental retardation, noncompliance, chemical dependencies (drug or alcohol), lack of adequate support system, personality disorders, underlying mental illness, and organic brain disorders [32].

Severe aortic regurgitation needs to be corrected simultaneously with LVAD placement to avoid a closed loop circulation between LV and the ascending aorta. In most cases, a bioprosthetic valve is placed. In some cases, the aortic valve is completely closed surgically which makes the patient very sensitive to any VAD malfunctioning [33]. Mitral surgery is only necessary for the presence of significant mitral stenosis compromising LV filling. Intra-cardiac shunts are typically closed at the time of VAD implantation. Pre-existing mechanical or biological mitral or aortic prosthetic valves usually do not cause complications during LVAD implantation [34,35].

Active infection is a contraindication for VADs. Bacterial infections are especially dangerous but on the other hand, a controlled infection like HIV may not be a contraindication to VADs [36-37].

Evolution of LVAD technology

**Figure 3:**
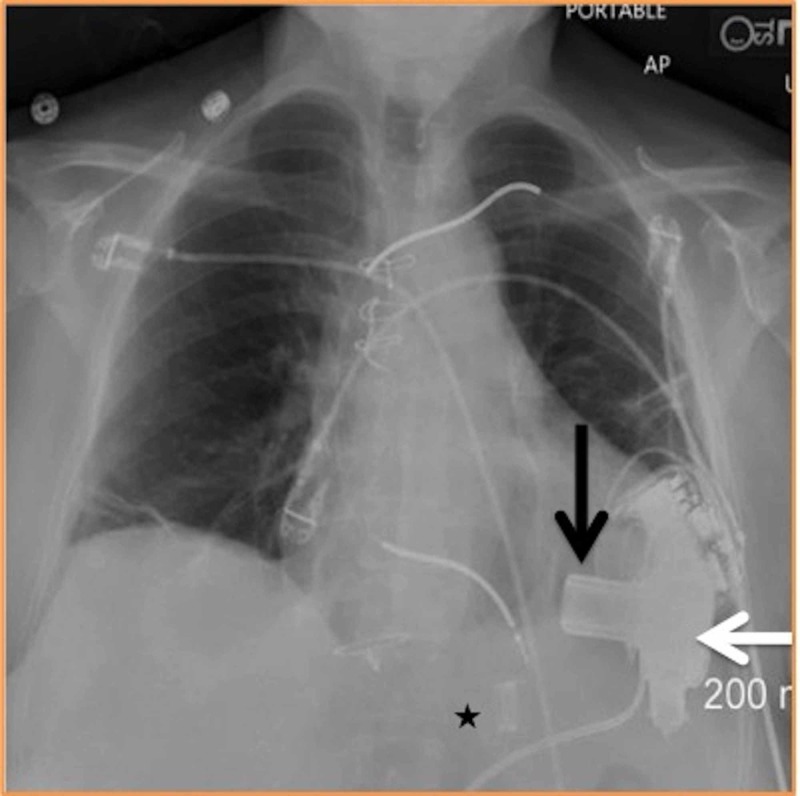
Plain radiograph “HVAD (HeartWare)” 3rd generation left ventricular assist device (LVAD). Black arrow – inflow cannula; white arrow – pump; black star – outflow cannula.

**Figure 4:**
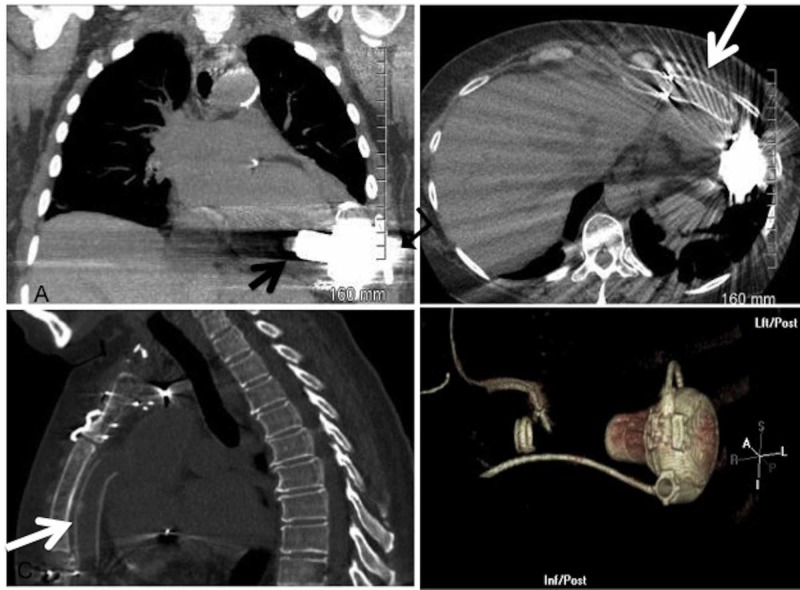
Coronal, axial, sagittal and volume-rendered 3D images showing 3rd generation left ventricular assist device (LVAD). Black arrow – inflow cannula; turn arrow – pump; white arrow – outflow cannula.

Mechanical circulatory devices (MCD) have been in use since the last three decades with initial devices trying to mimic natural (systolic and diastolic) hemodynamics of blood flow utilizing pneumatically driven left ventricular assist devices with unidirectional valves. Initial MCD liberated patients from extracorporeal membranous oxygenation until DT. In 2001, REMATCH trial was able to establish long-term suitability of MCD (HeartMate XVE) with 48% reduction in all-cause mortality (relative risk, 0.52; 95 percent confidence interval, 0.34 to 0.78; P = 0.001) for myocardial-replacement therapy in patients that were not eligible for heart transplant [38]. On the contrary, the device group showed twice the number of adverse events compared to medical therapy with the predominance of bleeding, infection and device malfunction. Clinical Utility Baseline Study (CUBS) and The Investigation of Non-transplant Eligible Patients Who Are Inotrope Dependent (INTREPID) trial showed some promising results in terms of survival with pulsatile MCDs, yet adverse events continued to be the limiting factor. This led to a paradigm shift as continuous flow MCDs reached the clinical arena. Solitary rotary component and smaller pump size in HeartMate II lead to limited wearing of mechanical parts, lower infection rate, enhanced placement within the body and axial flow through the device. Permanent magnetic field designed to rapidly spin a single impeller supported by mechanical, hydrodynamic or magnetic bearings leads to the development of contemporary second- and third-generation valveless LVAD. Second-generation axial pumps have the impeller outflow directed parallel to the axis of rotation. The rotor spins on mechanical (HeartMate II, Jarvik 2000, and HeartAssist5) or contact-free bearings (Incor, Berlin Heart, Berlin, Germany). Third-generation centrifugal pumps have the impeller outflow directed perpendicular from the axis of rotation (HeartWare Ventricular Assist Device [HVAD] and HeartMate III) (Figures 3, 4). Mixed design pumps, where blood flow follows the axis of rotation but exits perpendicular to the inflow (miniature ventricular assist device [MVAD] [HeartWare]), are also in use. Most recent pumps are contact-free, with no mechanical bearings (to avoid thrombus formation) and an impeller suspended using magnetic and/or hydrodynamic systems. Hydrodynamic levitation, in contact-free systems, uses a layer of blood (blood bearing) to lift the rotor (Incor, HVAD, and MVAD). Full magnetic levitation utilizes magnetic bearings only to levitate the rotor (HeartMate III).

In CF-LVADs, pump blood flow is directly proportional to rotor speed and inversely proportional to the pressure differential between the left ventricle and aorta (Figure 4). Axial flow pumps show a steep and inverse linear relationship between flow and head pressure. In contrast, this relationship is flatter and more susceptible to head pressure changes (i.e., more sensitive to re-load and afterload) in centrifugal pumps. With the same change in pressure, centrifugal pumps generate larger changes in flow, ranging from 0 to 10 l/min, whereas the axial pump flow ranges from 3 to 7 l/min. These hydrodynamic characteristics of centrifugal pumps lead to a more pulsatile waveform, better flow estimation, and a lower risk of suction events (e.g., in a setting of dehydration, arrhythmias, or right ventricular failure) [37,38].

Adverse outcomes and challenges with VADs

There is no doubt that LVADs have improved survival, functional capacity and QOL with advanced HF. However, only 30% of the recipients are free of any adverse effects within the first year [38-40]. The high burden of adverse effects associated with this therapy has been a limiting factor in offering it to patients without advanced HF. According to INTERMACS, the most common adverse effects (AE) (in declining frequency) include bleeding, infection, ventricular arrhythmias, respiratory failure and stroke [41].

AE of LVADs can be classified into three broad categories:

1. AE intrinsic to the pump and its constituents (pump malfunction, controller faults, driveline faults, and short-to-shield malfunction)

2. Patient-related AE (ventricular arrhythmias, valvular insufficiency, and RV failure)

3. AE resulting from pump patient interface (acquired von Willebrand disease, infection, stroke and pump thrombosis)

RV Dysfunction

Most patients with advanced HF have some degree of RV dysfunction. However severe RV dysfunction is often a contraindication for lifelong VAD therapy. RV failure, which is defined as a need for prolonged inotropic support or the temporary use of an RV assist device, occurs in 10-40% of LVAD implants and can lead to the longer hospital stay and a higher risk of perioperative death [42]. Late-onset RV failure has emerged as a new clinical challenge and associated with poor survival and decreased QOL [43].

Infections

Infection is now recognized as the leading cause of late mortality with an estimated prevalence of 8% and 18% at six months and 12 months respectively after diagnosis of driveline infection [41,44]. Most infections start as superficial driveline infections but can progress over months to become deep tissue infections [45,46]. The obligatory use of long-term antimicrobial therapy has led to the emergence of drug-resistant organisms such as Pseudomonas and Staphylococcus aureus.

LVADs and Ventricular Arrhythmias

Ventricular arrhythmias are common and frequently associated with increased mortality in patients with LVADs [47]. However, it is often suggested that sudden cardiac death is an uncommon mode of death in these patients. In some studies, patients with LVADs have been reported to survive for days to months despite being in rapid ventricular arrhythmias [48-50] and the postulated mode of death in these patients is primarily related to right heart failure and renal dysfunction.

The benefit of implantable cardioverter-defibrillators (ICDs) in patients with LVADs has remained unclear. Data for the effect of ICD on the survival of patients with LVADs have been conflicting and limited to observational studies with a smaller number of patients (12-17). However, in a recent meta-analysis of six observational studies [42-46], including 931 patients with LVADs, presence of an ICD was associated with a 39% relative risk reduction in all-cause mortality (RR: 0.61; 95% confidence interval [CI]: 0.46 to 0.82; p < 0.01). Among subgroup of patients with CF-LVAD (n = 361), ICD use was associated with a statistically nonsignificant trend toward improved survival (RR: 0.76; 95% CI: 0.51 to 1.12; p = 0.17).

Emerging role of imaging in LVAD

Echocardiography remains the cornerstone of imaging in LVAD and the role of computed tomography (CT) is rapidly emerging among patients that have poor echocardiographic windows. Recent guidelines endorse the important role of echocardiography in various stages in the clinical care of LVAD patients ranging from preoperative patient selection, perioperative imaging, postoperative surveillance, optimization of LVAD function, troubleshooting of LVAD alarms and evaluation of native myocardial recovery. There are recommendations and protocols for the timing and performance of echocardiography during LVAD patient selection, device implantation, and postoperative management [48-50]. Discussion regarding this is beyond the scope of the article and the readers can refer to the document from the American Society of Echocardiography for details regarding that.

Periodic LVAD surveillance trans-thoracic echocardiographic (TEE) exams are recommended to establish patient-specific baseline parameters for both LVAD and native heart function. It should be considered at approximately two weeks after device implantation or before index hospitalization discharge followed by surveillance TEE at one, three, six and 12 months post implantation and every six to 12 months thereafter.

Cardiac CT is rapidly emerging as an important modality of imaging in this group of patients and is usually a problem-solving tool when echocardiographic images are difficult or poor. There have been reports on the utility of MDCT (Multi-detector CT) for detection of complications where echocardiography has been unyielding [44-49]. There are also reports of the use of fluorodeoxyglucose/positron emission tomography (FDG-PET)/CT imaging for LVAD-related infections [49, 50].

## Conclusions

LVADs may prove to be a viable alternative to cardiac transplantation by providing long-term support without the major disabling AEs. With the ability to improve patient survival that is competitive with heart transplantation up to approximately two years, there has been dramatic improvement and progress of this therapeutic modality. While the focus on developing a more compatible device with improved durability and fewer AEs continues, the physicians taking care of this complex group of patients need to constantly keep themselves updated to ensure optimal care of the patients and recognize early signs of adverse effects and complications. This is especially true for primary care physicians/internists who are usually the “first contact” for a number of these patients. The future of fully implantable devices without the need for external driveline while reducing infection risk will also significantly improve QOL.
